# Time to Form a Habit: A Systematic Review and Meta-Analysis of Health Behaviour Habit Formation and Its Determinants

**DOI:** 10.3390/healthcare12232488

**Published:** 2024-12-09

**Authors:** Ben Singh, Andrew Murphy, Carol Maher, Ashleigh E. Smith

**Affiliations:** Alliance for Research in Exercise Nutrition and Activity (ARENA), Allied Health and Human Performance Academic Unit, University of South Australia, Adelaide, SA 5001, Australia; muraj009@mymail.unisa.edu.au (A.M.);

**Keywords:** habit formation, health behaviours, systematic review, automaticity, interventions

## Abstract

**Background:** Healthy lifestyles depend on forming crucial habits through the process of habit formation, emphasising the need to establish positive habits and break negative ones for lasting behaviour changes. This systematic review aims to explore the time required for developing health-related habits. **Methods:** Six databases (Scopus, PsychINFO, CINAHL, EMBASE, Medline and PubMed) were searched to identify experimental intervention studies assessing self-report habit or automaticity questionnaires (e.g., the self-report habit index (SRHI) or the self-report behavioural automaticity index (SRBAI)), or the duration to reach automaticity in health-related behaviours. Habit formation determinants were also evaluated. Meta-analysis was performed to assess the change in the SRHI or SRBAI habit scores between pre- and post-intervention, and the study quality was assessed using the PEDro scale. **Results:** A total of 20 studies involving 2601 participants (mean age range: 21.5–73.5 years) were included. Most studies had a high risk of bias rating (n = 11). Health behaviours included physical activity (n = 8), drinking water (n = 2), vitamin consumption (n = 1), flossing (n = 3), healthy diet (n = 8), microwaving a dishcloth (for foodborne disease reduction, n = 2) and sedentary behaviour reduction (n = 1). Four studies reported the median or mean times to reach habit formation, ranging from 59–66 days (median) and 106–154 days (means), with substantial individual variability (4–335 days). The meta-analysis showed significant improvements in habit scores pre- to post-intervention across different habits (standardised mean difference: 0.69, 95% CI: 0.49–0.88). Frequency, timing, type of habit, individual choice, affective judgements, behavioural regulation and preparatory habits significantly influence habit strength, with morning practices and self-selected habits generally exhibiting greater strength. **Conclusions:** Emerging evidence on health-related habit formation indicates that while habits can start forming within about two months, the time required varies significantly across individuals. A limitation of this meta-analysis is the relatively small number of studies included, with flossing and diet having the most evidence among the behaviours examined. Despite this, improvements in habit strength post-intervention are evident across various behaviours, suggesting that targeted interventions can be effective. Future research should aim to expand the evidence base with well-designed studies to better understand and enhance the process of establishing beneficial health habits.

## 1. Introduction

The promotion of health-related behaviours and the prevention of chronic diseases rely partly on the development and maintenance of health habits [1]. Habit formation is widely discussed across disciplines like psychology, behavioural science and public health as a crucial element for successful behaviour change interventions [2]. Research emphasises the importance of establishing healthful habits, ingraining behaviours into habitual patterns, and interrupting deeply embedded unhealthy habits to facilitate lasting modifications in behaviour [3,4]. The premise of habit formation involves the repetitive enactment of a behaviour within a consistent context, leading to its eventual automatic and effortless execution [2,5,6].

Healthy habit formation is a critical aspect of promoting long-term behaviour change and improving public health [7]. Automaticity is often used to represent successful ingrained habit formation. According to Bargh [8], automaticity is where a behaviour displays some or all the following features: lack of awareness, efficiency, uncontrollability and unintentionality. Lally and Gardner [7] proposed a four-stage framework for understanding habit formation: (1) deciding to take action, (2) translating intention into behaviour, (3) repeating the behaviour and (4) developing automaticity. This framework emphasises that while the first three stages are common to behaviour change in general, the development of automaticity is unique to habit formation, occurring through consistent repetition of a behaviour in a stable context over time [7]. Habit-based interventions have been proposed as a promising approach to promote long-lasting healthy behaviours, develop automaticity and prevent chronic diseases. Among the various factors influencing the habit formation process, dopamine, a chemical messenger that links actions and rewards, plays a crucial role [9]. Additionally, the concept of friction also significantly impacts habit formation by increasing or decreasing the resistance and effort associated with a behaviour [10,11]. By making behaviours easy and automatic, individuals can decrease obstacles and increase the likelihood of sustaining the habit long term.

A widely cited idea in popular culture, stemming from the book *Psycho-Cybernetics* by Maxwell Maltz, suggests that new habits can be formed with just 21 days of repetition [12]. However, this proposed timeline has been questioned, and the period necessary for the establishment of habits is thought to differ based on factors such as the specific behaviour and the individual [2]. The time and effort required for these behaviours to transform into ingrained habits likely varies widely, with multiple factors influencing the overall habit formation process. Practical strategies are believed to aid in the establishment of new healthy habits. One effective approach is to integrate novel habits with pre-existing ones, thereby creating a routine [2,13]. This entails linking a new habit to an existing one, simplifying integration into daily life. Initiating with modest, attainable and time-bound goals also enhances the probability of habit formation. Furthermore, the habit formation process thrives on consistency and repetition, as evidenced by studies indicating accelerated habit formation with increased practice frequency [2,5].

Previous research has aimed to investigate the complex relationships between intentions, habits and physical activity behaviour by evaluating moderating variables [14], examining the longitudinal association between habit strength and physical activity [15] and conducting meta-analyses of habit-behaviour relations [16]. Rhodes et al. [14], Hagger et al. [16] and Feil et al. [15] collectively highlight the complex interplay between intention, habit and physical activity, showing that specific sociodemographic and personality factors moderate the intention–physical activity relationship, habits independently predict behaviour and mediate past–future behaviour relations, and that the bidirectional relationship between habit and physical activity requires further differentiation between the instigation and execution of habits for better understanding and practical application. Building upon these findings, researchers have expanded their focus to explore the role of habit strength in a broader range of health behaviours and the potential of habit formation interventions.

Previous systematic reviews have used the self-report habit index (SRHI) [17] and self-report behavioural automaticity index (SRBAI) [18] to examine the associations between habit strength and health behaviours. The findings demonstrate that habit moderates the relationship between intention and behaviour for physical activity and nutrition [15,17]. Furthermore, a previous systematic review on the effects of habit formation interventions on physical activity habit strength found that habit formation interventions significantly increased physical activity habits (SMD = 0.31, 95% CI 0.14—0.48, *p* < 0.001) compared to the control groups [19]. Although these previous reviews have demonstrated the impact of habit strength on behaviour and the efficacy of habit formation interventions, their scope has been largely confined to physical activity, and to the authors’ knowledge, no review has evaluated the time required for the successful formation of individual virtuous health habits across a broader spectrum of health-related behaviours.

The development of new health habits is an important component of promoting healthy behaviours and preventing chronic diseases. The period necessary for the establishment of habits may differ based on both the specific behaviour and the individual. The time and effort required for these behaviours to transform into ingrained habits can vary widely, with multiple factors influencing the overall habit formation process. Therefore, this systematic review aims to investigate the time required for the development of health habits. In addition, it aimed to examine the factors (e.g., participant characteristics, health behaviour domain, the timing of practice and environmental factors) impacting the successful formation of health habits.

## 2. Materials and Methods

### 2.1. Registration and Protocol

The protocol for this systematic review was prospectively registered on PROSPERO (ID: CRD42023424694). This systematic review was conducted and reported in accordance with the Preferred Reporting Items for Systematic Reviews and Meta-Analyses (PRISMA) 2020 guidelines [20].

### 2.2. Information Sources and Search Strategy

Following several preliminary scoping searches, we employed a comprehensive search strategy to capture a broad array of studies pertinent to habit formation. Our objective was to include as many relevant experimental studies as possible that focused on the development of health habits. We refined our search criteria to include any study title that contained variations of the word ‘habit’ (i.e., ‘habit*’) along with terms indicative of the initiation or development of these habits, such as ‘form*’, ‘creat*’, ‘new’, ‘newly’ or ‘develop*’. This approach ensured the inclusion of studies addressing the creation or formation of new habits (see full details in Table S1). Six electronic databases were searched: Scopus, PsychINFO, CINAHL, EMBASE, Medline and PubMed. Searches included any language and were limited to humans, full-text peer-reviewed, and the year of publication from any time until the present, with the final search conducted on 18 October 2023.

### 2.3. Eligibility Criteria

Eligibility criteria were established using the Population, Intervention, Comparison, Outcomes and Study design (PICOS) framework [21], as follows: Population: Studies were included if they targeted adults over the age of 18 in the general population as well as clinical populations. Intervention: Published peer-reviewed articles investigating a new, virtuous health-related behaviour as an intervention were eligible. Studies needed to focus on habit development as the primary outcome and provide a clear operational definition of what constitutes a habit. Examples of possible virtuous health-related behaviours included but were not limited to exercise, diet, sleep, stress management or medication adherence. The studies aimed at ceasing a non-virtuous health-related behaviour, i.e., smoking or reducing social media use, were deemed ineligible. Comparator: No restrictions were placed on the inclusion of studies with or without a control or type of control group. Outcomes: The included studies were required to have an outcome that focused on the duration to reach habit formation (also referred to as automaticity). Measures could include self-reported questionnaires on achievement of automaticity, such as the SRHI [17], SRBAI [18] or the time that it took participants to reach their asymptote of an automaticity curve [22]. We used the original studies’ operational definitions of ‘habit formation’, acknowledging that habit formation occurs on a continuum. The studies used various thresholds to indicate ‘habit formation’, such as reaching the midpoint on habit strength scales or achieving a certain level of automaticity. Secondary outcomes included factors influencing habit formation duration. These factors may include participant characteristics (age and personality), health-related behaviour domains and intervention elements such as frequency, consistency of practice, timing of day or environmental cues. Studies not specifically investigating the time to achieve automaticity in forming new health habits or a change in them, but instead using habit in a general context (e.g., physical activity habits questionnaire), were also ineligible. Study Design: Peer-reviewed experimental or quasi-experimental studies focusing on habit formation were included. Conference papers were included if they provided full text, with abstracts being excluded. Common study designs included randomised control trials (RCTs), pre-post designs and quasi-experimental designs. Observational studies were excluded. Mixed methods studies were included if the quantitative component met the inclusion criteria.

### 2.4. Screening Procedure

**Figure 1:**
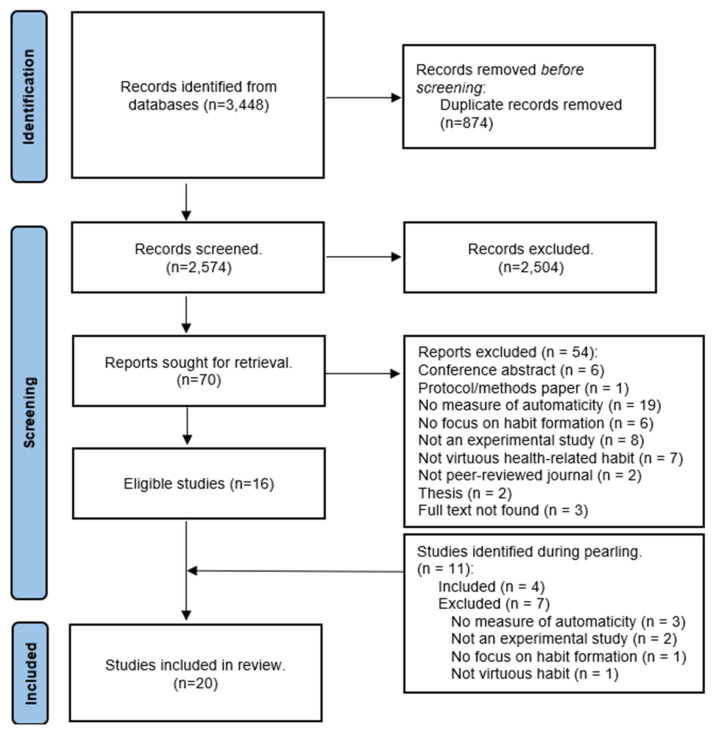
Screening overview.

Eligible studies were screened in Covidence based on the predetermined inclusion/exclusion criteria by two independent reviewers, with the discrepancies discussed until a consensus was formed. If a consensus was not achieved, a third independent reviewer decided upon its inclusion. Studies were first screened by title and abstract, with languages other than English being translated (n = 1). If no abstract was available, studies were obtained for a full-text review. Eligible studies were screened at the full-text stage by two independent reviewers to determine inclusion, with discrepancies discussed until a consensus was formed. If no consensus was reached, a third independent reviewer would decide upon inclusion. A summary of the screening process is highlighted in Figure 1. Further, the reference lists of the included studies were pearled for other potentially relevant articles through forward and backward pearling using artificial intelligence (AI) literature mapping software (version 2024) (LitMaps; Litmaps Ltd., Wellington, New Zealand).

### 2.5. Data Extraction

Data were extracted by two independent reviewers, with the discrepancies discussed until a consensus was reached. The extracted information included the study design and duration, participant characteristics (age, gender, population), targeted behaviour, intervention characteristics (e.g., duration, modality, components) and outcome measures (time to habit formation and determinants of habit formation).

### 2.6. Risk of Methodological Bias

Individual studies were assessed for methodological bias using the PEDro scale [23]. The scale consists of 11 criteria to evaluate if a clinical trial presents reliable and meaningful results for use in clinical practice [23]. Two independent reviewers assessed each individual study, with the discrepancies being resolved through discussion. Studies with a score of six or higher (of a possible score of 10; item one not contributing to the total score) were categorised as having a low risk of bias and lower than six as a high risk of bias [23].

### 2.7. Data Synthesis

In this systematic review, we employed both meta-analytical and narrative methods to synthesise the data from the included studies. Meta-analysis was performed to assess the change in the SRHI or SRBAI habit scores between pre- and post-intervention, which was analysed as a continuous outcome. Data were pooled using pre- and post-intervention means and standard deviations (SDs), and standardised mean differences (SMDs) were used as the effect measure to allow a comparison of data from different scales. For studies with multiple intervention arms, the number of participants in the control group was divided by the number of intervention arms for the purposes of the meta-analysis [24]. All meta-analyses were performed using RevMan software (version 5). Publication bias was not assessed because the meta-analysis included less than 10 unique studies [24]. For studies included multiple times in the meta-analysis, the sample size of the control group was divided accordingly to avoid double-counting [24]. Statistical heterogeneity was measured using Cochran’s Q test assessment, and the proportion of the overall outcome attributed to the variability was measured using the I^2^ statistic [24]. The following I^2^ cut-off values were used: 0 to 29% = no heterogeneity; 30 to 49% = moderate heterogeneity; 50 to 74% = substantial heterogeneity; and 75 to 100% = considerable heterogeneity [24]. The standardised classification effect sizes were 0.20 = small effect, 0.20 to 0.50 = medium effect and greater than 0.50 = large effect [25]. A *p*-value of < 0.05 was considered statistically significant.

In addition to the meta-analysis, we also conducted a narrative synthesis of the time taken to reach habit automaticity thresholds and the plateauing of automaticity scores over time. Finally, using the four-stage framework for habit formation proposed by Lally and Gardner [7], we synthesised the factors influencing the development of automaticity. Factors influencing progression through the four stages (deciding to take action, translating intention into behaviour, repeating the behaviour and developing automaticity) were categorised thematically across the studies, with a particular focus on the determinants of consistent repetition and automaticity development in stable contexts.

## 3. Results

### 3.1. Study Selection

A total of 3448 records were identified following a database search. After removing the duplicates, 2574 records remained and were screened based on their title and abstract. From this screening, 70 records were selected for a full-text review. After full-text screening, 16 studies met the eligibility criteria. Pearling (backward and forward citation tracking) of these included studies yielded an additional 11 studies, 4 of which were eligible. Therefore, a total of 20 studies were included in the final analysis (see Figure 1 for the reasons for exclusion). A list of excluded studies with reasons for exclusion is included in Table S2. The risk of bias, based on the PEDro scale, is shown in Table 1. Most studies had a high risk of bias rating (i.e., low quality, n = 11), while five had a low risk of bias rating (i.e., high quality).

### 3.2. Study Characteristics

A total of 2601 participants were included across all studies. The sample sizes ranged from 20 to 537 participants (median = 103), with the mean ages ranging from 21.5 to 73.5 years. An overview of the studies is shown in Table S3. One study recruited only females, while the remaining recruited both females and males. Most participants were recruited from universities (n = 6) [5,26,32,33,39,40] and the general public (n = 9) [22,28,30,34,35,36,38,41,42], with fewer studies recruiting hospital patients (n = 1) [29], older adults (n = 2) [31,43] and overweight or obese persons (n = 2) [27,37].

Approximately half the studies targeted a single behaviour (n = 12) [22,26,29,30,32,33,35,36,38,39,40,41], while the other half targeted multiple behaviours (n = 8) [5,27,28,31,34,37,42,43], two of which allowed participants to choose their target habits from a predefined list [5,42]. A range of health behaviours were targeted, including physical activity (n = 8) [5,27,28,29,31,35,37,42], drinking water (n = 2) [5,42], vitamin consumption (n = 1) [34], dental flossing (n = 3) [33,34,40], healthy nutrition behaviours (n = 8) [22,26,27,30,31,36,37,41], microwaving dishcloths (or sponge) to reduce the risk of foodborne disease (n = 2) [38,39] and sedentary behaviour (n = 1) [43].

The studies employed a range of delivery methods to support habit formation, including technology tools such as mobile apps and wearables (n = 2) [29,36]; text, email messaging or videos (n = 4) [29,39,41,42]; in-person or online counselling or workshops addressing planning, education, motivational enhancement or environmental modifications (n = 7) [22,28,31,33,35]; instructional/informational programs via posters, booklets leaflets or newsletters (n = 8) [26,27,28,29,33,34,37,43]; and planning activities like implementation intentions, self-monitoring or independently performing the behaviour (n = 9) [5,28,30,32,33,34,36,37,40].

Habit strength or automaticity scores were the primary outcome in most studies, assessed via the SRBAI (n = 9) [22,30,31,32,33,34,35,36,41], the SRHI (n = 10) [5,26,27,29,37,38,39,40,42,43] and the 12-item habit rater questionnaire n = 1 [28].

The study designs included randomised trials (n = 12) [22,27,28,31,32,35,37,38,39,40,41,43], a non-randomised controlled trial (n = 1) [33], single group pre-post studies (n = 6) [5,29,30,34,36,42] and mixed design (n = 1) [26], ranging from 3 weeks to 2 years. The studies varied in their approaches to examining habit formation and the duration of these examinations. Four studies specifically measured automaticity and recorded the time taken to achieve automaticity [5,22,32,37].

### 3.3. Time to Reach Habit Formation

Of the four studies reporting on the time to reach habit formation, Keller et al. [22] examined the formation of healthy eating habits and reported that habits were successfully formed in a median of 59 days (range: 4 to 335 days). However, only 23% of the included participants reached the predetermined threshold for habit formation. Similarly, Lally et al. [5] examined the formation of healthy eating, water consumption or exercise habits and reported that the median time to reach 95% automaticity was 66 days (range: 18 to 254 days). Lally et al.’s earlier [37] study focused on healthy eating and self-weighing and asked participants to self-report how long it took them to develop habits, with responses indicating a mean time of 91 days (SD = 55 days). Lastly, Fournier et al. [32] reported that forming the habit formation of daily stretching took a mean of 106 days and 154 days for morning and evening stretching, respectively.

### 3.4. Change in Habit Formation over Time

**Figure 2:**
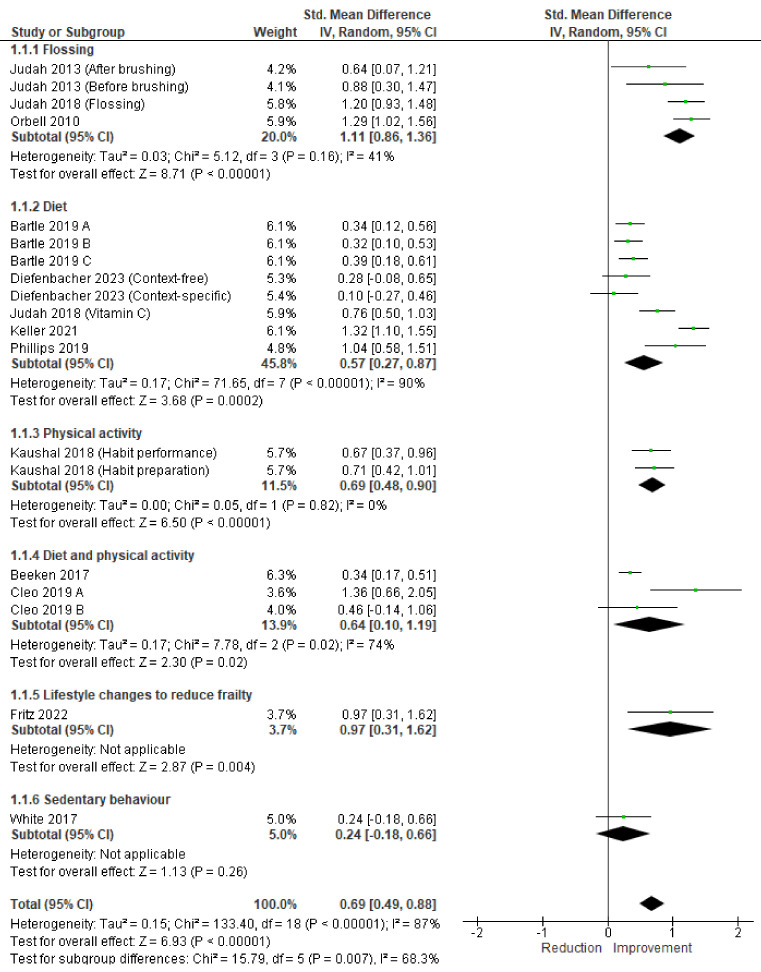
Meta-analysis results of change in habit scores between pre- and post-intervention. Note: (Bartle 2019 A [26]) fruit choice and poster; (Bartle 2019 B [26]) fruit choice and no poster; (Bartle 2019 C [26]) no fruit choice and poster; (Cleo 2019 A [28]) leaflet-based intervention; (Cleo 2019 B [28]) online intervention. Judah 2013 [27], Judah 2018 [34], Diefenbacher 2023 [33], Keller 2021 [22], Phillips, 2019 [39], Kaushal, 2018 [38], Beeken, 2017 [44], White, 2017 [42].

A meta-analysis was performed to assess the change in habit effect size between pre- and post-intervention for studies assessing the SRHI or SRBAI. Behaviours in these studies included diet [22,26,30], diet and/or physical activity [27,28,35,41], lifestyle changes to reduce frailty [26], sedentary behaviour [43], flossing [33,40] and flossing and Vitamin C consumption [34]. A pooled analysis of 12 studies (19 intervention conditions) showed a significant improvement (moderate-to-large effect size) in habit scores between pre- and post-intervention across the different interventions (SMD = 0.69, 95% CI = 0.49, 0.88; *I*^2^ *=* 87%, *p* < 0.01, Figure 2). The individual effect sizes for the different behaviours were SMD = 1.11 (95% CI = 0.86, 1.36; large effect size) for flossing, SMD = 0.57 (95% CI = 0.27, 0.87; moderate effect size) for diet, SMD = 0.69 (95% CI = 0.48, 0.90) for physical activity, SMD = 0.64 (95% CI = 0.10, 1.19; large effect size) for diet and physical activity, and SMD = 0.97 (SMD = 0.31, 1.62 large effect size) for lifestyle changes to reduce frailty. There were no significant effects for sedentary behaviour (SMD = 0.24, 95% CI = −0.18, 0.66).

### 3.5. Determinants Affecting Habit Formation

The determinants affecting habit formation were reported in twelve studies (60%).

Stage 1: Deciding to take action and translating intention into behaviour: The type of habit influenced the initial stages of habit formation. Lally et al. [5] and van der Weiden et al. [42] found that drinking water habits were stronger compared to habits related to exercise and fruit and vegetable consumption. The establishment of a regular dental flossing habit was influenced by both the intention to perform the behaviour and the specific plans made to implement that intention [40]. Habits chosen by individuals themselves led to stronger habit formation [42], suggesting that self-selection plays a role in the early stages of habit development.

Stage 2: Repeating the behaviour: Several factors influenced the repetition stage of habit formation. Fournier et al. [32] found that habits related to performing muscle stretches were stronger when practised in the morning compared to the evening, indicating that the timing of habit practice is important. Keller et al. [22] found no significant difference in the effectiveness of habit formation between habits triggered by time-based cues (e.g., drinking a glass of water at 9:00 a.m. each morning) and those by routine-based cues (e.g., drinking a glass of water after breakfast each morning). Individuals who exhibited a stable pattern of performing the target behaviour within a consistent context demonstrated stronger habit strengths [36], highlighting the importance of context stability in habit formation.

Lally et al. [5] further noted that early repetitions lead to larger increases in automaticity compared to later stages in the habit formation process, with a point where additional repetition no longer increases automaticity. Although consistency in repetition is crucial, the exact degree of required consistency remains unclear. Kilb et al. [36] suggested that context-dependent repetition can be enhanced through self-regulatory strategies, such as planning with implementation intentions or self-monitoring, which are commonly used in habit-based interventions and were included in our approach. Additionally, Kilb et al. and Lally et al. [5,36] emphasised the role of repetition as a critical determinant in habit formation, as outlined in our table. Repetition not only reinforces the behaviour but also helps to make it more automatic and deeply ingrained over time, further solidifying the habit.

Developing automaticity: The development of automaticity, the final stage unique to habit formation, was influenced by multiple factors. Kaushal et al. [35] found that affective judgement (i.e., enjoyment of the behaviour), behavioural regulation (i.e., planning specific details about the performance time and context of the behaviour) and preparatory habit (e.g., creating a daily exercise clothes routine) were significant mediators in physical activity habit formation, collectively mediating between the intervention and control groups and self-reported changes in MVPA.

Time to automaticity and intervention duration: The time taken to reach habit automaticity thresholds varied across studies. Lally et al. [5] observed score plateaus after rapid early gains, suggesting an initial acquisition phase before reaching automaticity. Across multiple studies (n = 8) [22,29,31,32,34,36,37,42], ongoing habit strengthening beyond one month of practice was reported. For example, Lally et al. [37] found that the average SRHI increases by 9 points after 32 weeks. Comparing Judah et al.’s [33,34] studies of 4- versus 16-week flossing interventions indicated that extended practice and counselling contact may bolster automaticity.

Interestingly, while the strength of habits varied across behaviours, Lally et al. [5] found that the duration required to achieve automaticity did not significantly differ between exercising, drinking water and eating fruit.

These findings highlight the complex interplay of factors influencing progression through the stages of habit formation, particularly in the development of automaticity through consistent repetition in stable contexts. The analysis of automaticity thresholds and score plateaus over time provides insights into the temporal aspects of habit formation across various behaviours and intervention durations.

## 4. Discussion

This systematic review set out to investigate the duration required for the development of health habits and identify the factors influencing the successful formation of these habits. Our analysis included 20 studies with a total of 2601 participants, examining a range of health-related behaviours such as physical activity, sedentary behaviour reduction, drinking water, healthy nutrition and dental flossing. We found that the time to reach habit formation varied widely, ranging from 59 to 66 days (median) and 106 to 154 days (means), with substantial individual variability (4 to 335 days) across different behaviours. The determinants affecting habit formation included the frequency and timing of practice, the type of habit and the context in which it was formed. Factors such as the intention to perform the behaviour, enjoyment of the behaviour, specific implementation plans, creating daily routines and the stability of the context in which the behaviour was performed influenced habit strength. The meta-analysis indicated a significant improvement in habit scores between pre- and post-intervention, demonstrating large effect sizes for flossing, diet and physical activity combined and lifestyle changes to reduce frailty and moderate effect sizes for diet.

To our knowledge, this is the first systematic review to examine the duration of habit formation and the factors influencing it across a broad range of health habits. Our findings both confirm and extend those of Gardner, Rebar and Lally [44], who conducted a narrative synthesis of five studies examining dietary habits (n = 1), physical activity (n = 1), combined dietary and physical activity (n = 1), media use (n = 1) and self-chosen health behaviours (n = 1). These studies explored habit formation and the potential moderators over periods ranging from 30 to 90 days, employing measures of behaviour frequency and habit development, primarily focusing on researcher-prompted attempts and the effects of missed performances. In contrast, our meta-analysis of the changes in habit scores from intervention studies reveals an overall effect size of 0.67, with individual behaviour effect sizes ranging from 0.56 to 1.11 for various health-related behaviours, providing valuable insights into the impact of interventions on habit development over time. We adhered to the highest quality systematic review methodology, including undertaking a comprehensive search strategy across six major electronic databases (Scopus, PsychINFO, CINAHL, EMBASE, Medline and PubMed) along with forward and backward pearling, the use of the PICOS framework for defining eligibility criteria allowing for a clear and systematic selection process and the use of independent screening and data extraction by two reviewers. Furthermore, we were able to employ a meta-analytical approach to quantitatively synthesise the habit formation data, offering insights into the effect sizes of various interventions for different health habits.

Despite its strengths, this systematic review also has several limitations. In searching for relevant studies, we found it somewhat challenging to distinguish between studies that truly focused on habit formation and those that used the term “habit” more generally in the context of health behaviour. For example, one such study focusing on infant feeding behaviour was excluded from the final review as the authors felt it was an exploratory study embedded in a larger RCT and was not focused on individual habit formation but rather reliant on the behaviour of the infant. This challenge was overcome through extensive team discussions over an extended period, preliminary literature searches to identify examples of relevant and irrelevant studies and the development of tightly defined inclusion and exclusion criteria that were developed at the protocol registration stage. Only a modest evidence base was identified for inclusion in the review based on our strict criteria, comprising 20 studies, a third of which lacked control groups, and most had relatively small sample sizes (n < 100). The included studies were heterogeneous in nature in terms of their design, targeted behaviours, intervention methods, outcome measures and assessment time frames, making synthesis challenging. The small number of studies that focused explicitly on the duration of habit formation and variability in their study designs prevented us from conducting a meta-analysis for the duration to reach automaticity. However, we were able to undertake narrative syntheses that addressed the study’s aims regarding the duration of habit formation and the factors affecting habit formation. Further, a limitation of our review is that only four studies directly reported on the requirements for developing health habits. The remaining 16 studies, while focused on pre- and post-changes in habits, provided valuable data on how factors like the intervention type, intensity and participant characteristics influence habit development over time. This apparent scarcity of studies directly measuring the habit formation time highlights a significant gap between popular advice and scientific evidence in this field, pointing to an important area for future research.

There was a wide range of durations required for the formation of health habits. For example, the median and mean habit acquisition times ranged between 59 and 66 days and 106 and 154 days, respectively. In addition, substantial variance emerged at the individual level, with the durations for individuals to form habits ranging from 4 to 335 days [5,22]. In summary, our results here, along with findings from others [5], clearly refute the popular notion that habits can be formed in approximately 21 days [45]. This is likely dependent on not only personal factors but also the habit you are trying to form [46]. While we considered guidance documents from experts such as Gardner and colleagues [44], the complexity and heterogeneous nature of habit research meant that identifying relevant studies remained challenging. We addressed this through extensive team discussions, preliminary literature searches to identify examples of relevant and irrelevant studies and the development of tightly defined inclusion and exclusion criteria at the protocol registration stage. We defined a ‘habit formation’ study as one that tracks the development of habitual behaviour over time, emphasising how repetition in a consistent context contributes to habit formation. Additionally, we required these studies to employ reliable measurement tools and follow participants over a sufficient duration to capture the potential changes effectively. However, the diverse approaches to studying habits and the nuanced nature of habit formation meant that applying these criteria often required careful consideration and discussion among our team.

The variability in habit formation durations observed in our review may be attributed to several factors. The nature of the behaviour itself is a critical determinant; simpler, repetitive behaviours with clear cues and immediate rewards are easier to automate [46]. Additionally, the context in which the behaviour is practised plays an important role. Habits formed in stable, consistent environments are more likely to become automatic. Furthermore, personal characteristics, such as individual motivation, pre-existing routines and the presence of supportive social and environmental factors, also influence the speed of habit formation. These findings align with a recent review article by Albarracín et al. [47] that synthesises multidisciplinary meta-analyses on behavioural determinants, which similarly found that individual interventions have varying impacts.

Our systematic review identified several key determinants that appear to influence the successful formation of health habits. Albarracín et al.’s review [47] highlighted those interventions addressing habits and behavioural attitudes as being more effective than those targeting knowledge and general attitudes [47]. Our review extends these findings by providing specific insights into the factors that enhance successful habit formation. In particular, practising habits in the morning appears to lead to enhanced habit formation compared to practising in the evening [32]. Contextual factors and personal motivations are also important. The studies by Judah et al. [33] on dental flossing and Keller et al. [22] on healthy eating habits highlight the importance of specific implementation plans and the stability of the context in which the behaviour is performed. Furthermore, van der Weiden et al. [42] found that habits that were self-selected were more successful than those that were assigned without the participants’ choice. These findings align with broader theories of health behaviours, such as the theory of planned behaviour [48], which posits that intention and perceived behavioural control are key predictors of behaviour, and self-determination theory, which emphasises the role of autonomy, competence and relatedness in fostering intrinsic motivation and sustained behaviour change [49].

Our meta-analysis revealed significant improvements in the habit scores between pre- and post-intervention, with large effect sizes observed in descending order for flossing, lifestyle changes to reduce frailty, combined diet and physical activity interventions, physical activity interventions and diet interventions. The overall meta-analysis of the change in habit scores between pre- and post-intervention across the various interventions produced an SMD of 0.69, suggesting that interventions focusing on habit formation are highly effective. This effect size is considerably larger than that reported in a previous systematic review by Feil and colleagues [15], investigating the effects of habit formation interventions on physical activity habit strength (SMD = 0.31). Interestingly, many studies included in Feil’s review were not eligible for our review, likely due to our strict requirement for the included studies to focus on habit formation. Thus, the differences in effect sizes between our reviews may reflect differences in the efficacy of interventions with an explicit focus on habit formation versus those focused on behaviour change more generally. Alternatively, the larger effect size observed in our study may be due to the wider range of behaviours included in our meta-analysis (diet, physical activity, lifestyle changes to reduce frailty, flossing and Vitamin C consumption), compared with just physical activity in the previous study [15]. In particular, our findings highlighted that some health behaviours may be easier to form a habit than others. In particular, there was a pattern for simpler, repetitive behaviours with clear cues and immediate rewards (such as flossing and drinking water) to show large effects, while more complex behaviours, such as regular healthy eating and physical activity, showed somewhat smaller effects. Overall, this meta-analysis revealed significant improvements in the habit scores between pre- and post-intervention across various health behaviours. While we observed differences in the effect sizes across behaviours, it is important to note that these differences may reflect variations in intervention effectiveness rather than inherent differences in habit formation potential across behaviours.

Determining an average duration for habit formation is important, as it helps set realistic expectations for individuals and practitioners, enhances motivation for behaviour change, and allows researchers to identify common patterns that inform targeted strategies across various contexts. The findings of this systematic review have important implications for individuals, health practitioners and public health initiatives. Brief 21-day challenges or kickstarts are unlikely to be sufficient to firmly ingrain new habits. Individuals should anticipate a timeframe of at least two to five months to develop automaticity in new health habits, rather than the commonly cited 21-day period. Having a realistic expectation that habit formation takes time can help individuals stay motivated and persist through the initial stages of behaviour change. For health practitioners, interventions should be designed with sufficient duration and ongoing support to help individuals reach and maintain automaticity, particularly for complex behaviours like physical activity and healthy eating. Strategies such as morning practices, self-selection of habits and integration into stable daily routines may increase success. Public health programs should prioritise simpler, repetitive behaviours with clear cues and immediate rewards to achieve quick wins and build momentum for more complex behaviour changes. Additionally, policies should support long-term interventions and provide resources for sustained behaviour change efforts, emphasising the importance of realistic timelines.

Our systematic review highlighted a surprising scarcity of experimental evidence focused on the duration of habit formation and the factors that influence it. This lack of rigorous studies is in stark contrast to the widespread perception of habits as central to a healthy lifestyle and the extensive, conventional wisdom surrounding habit formation. The eligibility criteria for our study intentionally encompassed studies using habit-based interventions, allowing us to capture a broader understanding of how habit principles are applied in behaviour change contexts, providing insights that extend beyond the limited number of studies strictly defined as ‘habit formation’ research. While Gardner et al. [44] emphasised the formation of entirely new cue-behaviour associations as central to habit formation, our study intentionally included habit-based interventions to capture a broader application of habit-forming techniques. This approach acknowledges that while some interventions may reinforce existing behaviours rather than create new ones, they still leverage principles of habit formation that can support sustained behaviour change. By examining studies that apply habit principles more generally, our review provides insights into the diverse ways habit-based strategies can be employed to encourage both new and maintained behaviours, offering a nuanced understanding of habit principles beyond the formation of strictly new habits. Although some overlap between habit responsiveness and intervention effectiveness may occur, our review captures the real-world application of habit-based strategies, offering insights into how habit strength responds within these intervention contexts. To advance understanding in this field, there is a pressing need for large, high-quality studies that track habit formation over extended periods and map the trajectory of automaticity. Future research should include diverse populations and a broad range of health behaviours to ensure generalisability. Importantly, these studies should incorporate objective measures of behaviour practice rather than relying solely on self-reported outcomes to enhance the reliability and validity of the findings. By addressing these gaps, future research can provide more definitive insights into the processes and timelines of habit formation, ultimately informing the development of more effective health interventions.

### Implications

The findings of this systematic review and meta-analysis have significant implications for both individuals and health practitioners seeking to develop health habits. Contrary to the widely held belief that habits can form within 21 days, our research indicates that habit formation typically requires a duration of 2 to 5 months for most health behaviours to become automatic. This insight is critical, as it sets more realistic expectations for individuals attempting lifestyle changes.

For individuals, recognising that health habits take time to form can help sustain motivation through the early stages of behaviour change. Short-term “21-day challenges” are often insufficient for lasting habits, especially for complex behaviours like exercise and healthy eating. Consistent practice over several months is needed for habit formation. For health practitioners, these findings suggest that interventions should be longer and offer ongoing support. Strategies like morning routines, allowing choice, and embedding habits into daily routines may improve success. Practitioners should counsel patients on the extended timeframes for habit formation and provide tools like self-monitoring and accountability. Public health programs should emphasise sustained efforts beyond short-term interventions, with policies supporting long-term behaviour change. Targeting simple, repetitive behaviours with clear cues and immediate rewards can create early wins, building momentum for more challenging habits. Lastly, future research should focus on longer follow-up periods in habit intervention studies, as shorter studies may underestimate the time needed for habits to develop. Shifting toward extended timelines and realistic expectations can promote more sustainable behaviour change.

## 5. Conclusions

This systematic review of 20 studies involving 2601 participants challenges the prevailing notion of rapid habit formation, revealing that health-related habits typically require 2–5 months to develop, with substantial individual variability ranging from 4 to 335 days. The meta-analysis demonstrated significant improvements in habit scores across various health behaviours, with key determinants including morning practices, personal choice, and behavioural characteristics. Findings emphasise the need for healthcare practitioners to design interventions with extended timelines and sustained support, moving beyond short-term challenges to facilitate meaningful behaviour change. By providing a nuanced understanding of habit formation, this review offers critical insights for developing more effective strategies in promoting long-term health behaviours, highlighting the importance of realistic expectations, consistent practice, and personalised approaches in supporting individuals’ lifestyle modifications.

## Figures and Tables

**Table 1:** Study quality assessments using the PEDro scale (n = 20).

Author, Year	Eligibility Criteria	Random Subject Allocation	Concealed Allocation	Baseline Group Similarity	Blinding of Subjects	Blinding of Therapists	Blinding of Assessors	Outcome Measures	Intention-to-Treat	Between-Group Comparison	Point and Variability Data	Overall Score
Bartle 2019 [26]	**×**	•	•	NA	•	**×**	**×**	•	•	•	•	7 (High)
Beeken, 2017 [27]	•	•	•	•	**×**	**×**	•	**×**	•	•	•	7 (High)
Cleo 2019 [28]	•	•	•	•	**×**	**×**	•	•	•	•	•	8 (High)
Davis 2020 [29]	**×**	**×**	NA	NA	**×**	**×**	**×**	•	•	•	**×**	3 (Low)
Diefenbacher 2023 [30]	•	**×**	NA	NA	**×**	**×**	**×**	**×**	•	•	•	3 (Low)
Fritz 2022 [31]	•	•	•	•	•	**×**	•	**×**	•	•	•	8 (High)
Fournier 2017 [32]	•	•	**×**	•	**×**	**×**	**×**	•	•	**×**	•	5 (Low)
Judah 2013 [33]	•	**×**	NA	•	**×**	**×**	**×**	•	•	•	•	5 (Low)
Judah 2018 [34]	**×**	**×**	NA	NA	**×**	**×**	**×**	•	•	•	•	4 (Low)
Kaushal, 2018 [35]	**×**	•	**×**	•	**×**	**×**	**×**	•	•	•	•	6 (High)
Keller 2021 [22]	•	•	**×**	•	**×**	**×**	**×**	**×**	•	•	•	5 (Low)
Kilb 2022 [36]	•	**×**	NA	NA	**×**	**×**	**×**	**×**	•	•	**×**	2 (Low)
Lally 2008 [37]	•	•	**×**	**×**	**×**	**×**	**×**	•	•	•	•	5 (Low)
Lally 2010 [5]	**×**	**×**	NA	NA	**×**	**×**	**×**	•	•	•	**×**	3 (Low)
Mergelsberg 2021 [38]	**×**	•	**×**	•	**×**	**×**	**×**	**×**	•	•	•	5 (Low)
Mullan 2014 [39]	•	•	•	•	**×**	**×**	**×**	•	•	•	•	7 (High)
Orbell 2010 [40]	**×**	•	**×**	•	**×**	**×**	**×**	•	•	•	•	6 (High)
Phillips, 2019 [41]	•	•	**×**	•	**×**	**×**	•	•	•	•	•	7 (High)
Van der Weiden 2020 [42]	•	**×**	NA	NA	**×**	**×**	**×**	**×**	•	•	•	3 (Low)
White, 2017 [43]	•	•	•	•	•	**×**	**×**	•	•	•	•	8 (High)

PEDro scale items: 1. Eligibility criteria were specified; 2. subjects were randomly allocated to groups; 3. allocation was concealed; 4. the groups were similar at baseline regarding the most important prognostic indicators; 5. there was blinding of all subjects; 6. there was blinding of all therapists who administered the therapy; 7. there was blinding of all assessors who measured at least one key outcome; 8. measures of at least one key outcome were obtained from more than 85% of the subjects initially allocated to groups; 9. all subjects for whom outcome measures were available received the treatment or control condition as allocated or, where this was not the case, data for at least one key outcome were analysed by “intention to treat”; 10. the results of between-group statistical comparisons are reported for at least one key outcome; 11. the study provides both point measures and measures of variability for at least one key outcome. A score of 6 or higher was considered high quality, and trials receiving less than 6 were classified as low quality. Item 1 does not count towards the overall score. **×** = no, • = yes, NA = not applicable.

## Data Availability

All data are included in the manuscript and Supplementary Materials.

## Supplementary Materials

The following supporting information can be downloaded at: https://www.mdpi.com/article/10.3390/healthcare12232488/s1, Table S1: Search strategy; Table S2: List of full-text exclusions; Table S3: Overview of included studies.

## References

[1] Gardner B., Arden M.A., Brown D., Eves F.F., Green J., Hamilton K., Hankonen N., Inauen J., Keller J., Kwasnicka D. (2023). Developing habit-based health behaviour change interventions: Twenty-one questions to guide future research. Psychol. Health.

[2] Gardner B., Lally P., Wardle J. (2012). Making health habitual: The psychology of ‘habit-formation’ and general practice. Br. J. Gen. Pract..

[3] Zhang C., Adriaanse M.A., Potgieter R., Tummers L., de Wit J., Broersen J., de Bruin M., Aarts H. (2022). Habit formation of preventive behaviours during the COVID-19 pandemic: A longitudinal study of physical distancing and hand washing. BMC Public Health.

[4] Gardner B. (2015). A review and analysis of the use of ‘habit’ in understanding, predicting and influencing health-related behaviour. Health Psychol. Rev..

[5] Lally P., van Jaarsveld C.H.M., Potts H.W.W., Wardle J. (2010). How are habits formed: Modelling habit formation in the real world. Eur. J. Soc. Psychol..

[6] Gardner B., Rebar A.L. (2019). Habit Formation and Behavior Change.

[7] Lally P., Gardner B. (2013). Promoting habit formation. Health Psychol. Rev..

[8] Bargh J.A. (1994). The four horsemen of automaticity: Awareness, intention, efficiency, and control in social cognition. Handbook of Social Cognition: Basic Processes; Applications.

[9] Wickens J.R., Horvitz J.C., Costa R.M., Killcross S. (2007). Dopaminergic mechanisms in actions and habits. J. Neurosci..

[10] Verplanken B., Orbell S. (2022). Attitudes, Habits, and Behavior Change. Annu. Rev. Psychol..

[11] Groot Kormelink T. (2023). How People Integrate News into Their Everyday Routines: A Context-Centered Approach to News Habits. Digit. Journal..

[12] Maltz M., Furey M. (2022). Psycho-Cybernetics.

[13] Lally P., Wardle J., Gardner B. (2011). Experiences of habit formation: A qualitative study. Psychol. Health Med..

[14] Rhodes R.E., Cox A., Sayar R. (2021). What Predicts the Physical Activity Intention–Behavior Gap? A Systematic Review. Ann. Behav. Med..

[15] Feil K., Allion S., Weyland S., Jekauc D. (2021). A Systematic Review Examining the Relationship Between Habit and Physical Activity Behavior in Longitudinal Studies. Front. Psychol..

[16] Hagger M., Hamilton K., Phipps D., Protogerou C., Zhang C.-Q., Girelli L., Mallia L., Lucidi F. (2023). Effects of Habit and Intention on Behavior: Meta-Analysis and Test of Key Moderators. Motiv. Sci..

[17] Gardner B., de Bruijn G.J., Lally P. (2011). A systematic review and meta-analysis of applications of the Self-Report Habit Index to nutrition and physical activity behaviours. Ann. Behav. Med..

[18] Gardner B., Abraham C., Lally P., de Bruijn G.-J. (2012). Towards parsimony in habit measurement: Testing the convergent and predictive validity of an automaticity subscale of the Self-Report Habit Index. Int. J. Behav. Nutr. Phys. Act..

[19] Ma H., Wang A., Pei R., Piao M. (2023). Effects of habit formation interventions on physical activity habit strength: Meta-analysis and meta-regression. Int. J. Behav. Nutr. Phys. Act..

[20] Page M.J., McKenzie J.E., Bossuyt P.M., Boutron I., Hoffmann T.C., Mulrow C.D., Shamseer L., Tetzlaff J.M., Akl E.A., Brennan S.E. (2021). The PRISMA 2020 statement: An updated guideline for reporting systematic reviews. BMJ.

[21] Mehrdad A.-B., Ali J. (2020). Population, Intervention, Comparison, Outcomes and Study (PICOS) design as a framework to formulate eligibility criteria in systematic reviews. Emerg. Med. J..

[22] Keller J., Kwasnicka D., Klaiber P., Sichert L., Lally P., Fleig L. (2021). Habit formation following routine-based versus time-based cue planning: A randomized controlled trial. Br. J. Health Psychol..

[23] de Morton N.A. (2009). The PEDro scale is a valid measure of the methodological quality of clinical trials: A demographic study. Aust. J. Physiother..

[24] Chandler J., Cumpston M., Li T., Page M.J., Welch V.J.H.W. (2022). Cochrane Handbook for Systematic Reviews of Interventions Version 6.3 (Updated February 2022). https://training.cochrane.org/handbook.

[25] Cohen J. (1988). Statistical Power Analysis for the Behavioral Sciences.

[26] Bartle T., Mullan B., Novoradovskaya E., Allom V., Hasking P. (2019). The role of choice in eating behaviours. Br. Food J..

[27] Beeken R.J., Leurent B., Vickerstaff V., Wilson R., Croker H., Morris S., Omar R.Z., Nazareth I., Wardle J. (2017). A brief intervention for weight control based on habit-formation theory delivered through primary care: Results from a randomised controlled trial. Int. J. Obes..

[28] Cleo G., Glasziou P., Beller E., Isenring E., Thomas R. (2019). Habit-based interventions for weight loss maintenance in adults with overweight and obesity: A randomized controlled trial. Int. J. Obes..

[29] Davis J.W. (2020). Physical activity habit formation through a technology-based program. J. Am. Assoc. Nurse Pract..

[30] Diefenbacher S., Lally P., Gardner B. (2023). Habit formation in context: Context-specific and context-free measures for tracking fruit consumption habit formation and behaviour. Br. J. Health Psychol..

[31] Fritz H., Hu Y.-L. (2022). Habit Formation Intervention to Reduce Frailty Risk Factors: A Feasibility Study. Am. J. Occup. Ther..

[32] Fournier M., d’Arripe-Longueville F., Rovere C., Easthope C.S., Schwabe L., El Methni J., Radel R. (2017). Effects of circadian cortisol on the development of a health habit. Health Psychol..

[33] Judah G., Gardner B., Aunger R. (2013). Forming a flossing habit: An exploratory study of the psychological determinants of habit formation. Br. J. Health Psychol..

[34] Judah G., Gardner B., Kenward M.G., DeStavola B., Aunger R. (2018). Exploratory study of the impact of perceived reward on habit formation. BMC Psychol..

[35] Kaushal N., Rhodes R.E., Meldrum J.T., Spence J.C. (2018). Mediating Mechanisms in a Physical Activity Intervention: A Test of Habit Formation. J. Sport. Exerc. Psychol..

[36] Kilb M., Labudek S. (2022). Effects of behavioral performance, intrinsic reward value, and context stability on the formation of a higher-order nutrition habit: An intensive longitudinal diary study. Int. J. Behav. Nutr. Phys. Act..

[37] Lally P., Chipperfield A., Wardle J. (2008). Healthy habits: Efficacy of simple advice on weight control based on a habit-formation model. Int. J. Obes..

[38] Mergelsberg E.L.P., Mullan B.A., Allom V., Scott A. (2021). An intervention designed to investigate habit formation in a novel health behaviour. Psychol. Health.

[39] Mullan B., Allom V., Fayn K., Johnston I. (2014). Building habit strength: A pilot intervention designed to improve food-safety behavior. Food Res. Int..

[40] Orbell S., Verplanken B. (2010). The automatic component of habit in health behavior: Habit as cue-contingent automaticity. Health Psychol..

[41] Phillips L.A., Johnson M., More K.R. (2019). Experimental test of a planning intervention for forming a ‘higher order’ health-habit. Psychol. Health.

[42] Van der Weiden A., Benjamins J., Gillebaart M., Ybema J.F., De Ridder D. (2020). How to form good habits? A longitudinal field study on the role of self-control in habit formation. Front. Psychol..

[43] White I., Smith L., Aggio D., Shankar S., Begum S., Matei R., Fox K.R., Hamer M., Iliffe S., Jefferis B.J. (2017). On Your Feet to Earn Your Seat: Pilot RCT of a theory-based sedentary behaviour reduction intervention for older adults. Pilot. Feasibility Stud..

[44] Gardner B., Rebar A.L., Lally P. (2022). How does habit form? Guidelines for tracking real-world habit formation. Cogent Psychol..

[45] Gardner B. (2021). Busting the 21 Days Habit Formation Myth.

[46] Verplanken B. (2018). The Psychology of Habit: Theory, Mechanisms, Change, and Contexts.

[47] Albarracín D., Fayaz-Farkhad B., Granados Samayoa J.A. (2024). Determinants of behaviour and their efficacy as targets of behavioural change interventions. Nat. Rev. Psychol..

[48] Armitage C.J., Conner M. (2001). Efficacy of the theory of planned behaviour: A meta-analytic review. Br. J. Soc. Psychol..

[49] Deci E.L., Ryan R.M. (2012). Self-determination theory. Handbook of Theories of Social Psychology.

