# Supplementary material for: Time to Form a Habit: A Systematic Review and Meta-Analysis of Health Behaviour Habit Formation and Its Determinants
Source: Healthcare (Basel). 2024 Dec 9;12(23):2488. doi: 10.3390/healthcare12232488 (PMC11641623; doi:10.3390/healthcare12232488)
Supplement: Supplementary file 1 [file healthcare-12-02488-s001.zip › healthcare-3218243-supplementary.pdf]

Table S1. Search strategy

Database	Search Strategy
Medline	(((habit OR habits OR habitua*) AND (health* OR disease* OR wellbe* OR prevent* OR lifestyle*)) adj4 (form* OR creat* OR establish* OR develop*)) AND Publication Type[pt]
Scopus	(((habit OR habits OR habitua*) AND (health* OR disease* OR wellbe* OR prevent* OR lifestyle*)) w/4 (form* OR creat* OR establish* OR develop*)) AND (LIMIT-TO(SUBAREA, "MEDICINE"))
PsychINFO	((habit OR habits OR habitua*) AND (health* OR disease* OR wellbe* OR prevent* OR lifestyle*)) adj4 (form* OR creat* OR establish* OR develop*)) AND (subject field (Psychology OR Behavioral Science))
CINAHL	(((habit OR habits OR habitua*) AND (health* OR disease* OR wellbe* OR prevent* OR lifestyle*)) adj4 (form* OR creat* OR establish* OR develop*)) AND (MH "Health Behavior")
EMBASE	(((habit OR habits OR habitua*) AND (health* OR disease* OR wellbe* OR prevent* OR lifestyle*)) adj4 (form* OR creat* OR establish* OR develop*)) AND (publication type article/full text)
PubMed	(((habit OR habits OR habitua*) AND (health* OR disease* OR wellbe* OR prevent* OR lifestyle*)) adj4 (form* OR creat* OR establish* OR develop*)) AND Publication Type[pt]

Table S2. List of full-text exclusions.

	Author	Date	Title	Reason
1	Valenzuela-Beltrán, M.; Andrade, Á G.; Stawarz, K.; Rodríguez, M. D.	2022	A Participatory Sensing Study to Understand the Problems Older Adults Faced in Developing Medication-Taking Habits	No measure of automaticity
2	Nalbantski, B.; Gyurov, O.	1975	A study on the degree of health education and formed habits on the questions about personal hygiene, smoking and usage of alcohol in a group of young servicemen (Bulgarian)	Article not found
3	Phillips et al.	2013	Assessing theoretical predictors of long-term medication adherence: Patients' treatment-related beliefs, experiential feedback and habit development	Not experimental design
4	Beeken, R. J.; Vickerstaff, V.; Leurent, B.; Omar, R.; Croker, H.; Morris, S.; Nazareth, I.; Wardle, J.	2015	Associations between habit formation and weight loss in the 10 top tips (10tt) trial: A randomised controlled trial of habit based advice for weight control in general practice	Conference abstract
5	Carrera, M.; Royer, H.; Stehr, M.; Sydnor, J.	2018	Can financial incentives help people trying to establish new habits? Experimental evidence with new gym members	No measure of automaticity
6	Bai, Y.; Xu, B.; Jiang, S.; Yang, H.; Cui, J.	2013	Can you form healthy habit? Predicting habit forming states through mobile phone	No measure of automaticity
7	Haerana, B. T.; Jalaluddin, S.; Satrianegara, M. F.; Wijaya, D. R.; Basri, S.; Bujawati, E.	2021	COVID-19 exposure risk level in daily activity in adapting to new habits in the pandemic period	Not virtuous health-related habit
8	Beshears, J.; Lee, H. N.; Milkman, K. L.; Mislavsky, R.; Wisdom, J.	2021	Creating exercise habits using incentives: The trade-off between flexibility and routinization	No measure of automaticity
9	Lee Mi, Sook; Gu Mee, Ock	2020	Development and Effects of Combined Exercise and an Exercise Habit Formation Program for Undergraduate Nursing Students	No focus on habit formation

10	McCrum, L. A.; Watson, S.; McGowan, L.; McGuinness, B.; Cardwell, C.; Clarke, M.; Woodside, J. V.; McKenna, G.	2020	Development and feasibility of a tailored habit-based dietary intervention coupled with natural tooth replacement on the nutritional status of older patients	No measure of automaticity
11	Kliemann, N.; Croker, H.; Johnson, F.; Beeken, R. J.	2019	Development of the Top Tips Habit-Based Weight Loss App and Preliminary Indications of Its Usage, Effectiveness, and Acceptability: Mixed-Methods Pilot Study	No measure of automaticity
12	Ahlgren, C.; Hammarström, A.; Sandberg, S.; Lindahl, B.; Olsson, T.; Larsson, C.; Fjellman-Wiklund, A.	2016	Engagement in New Dietary Habits—Obese Women's Experiences from Participating in a 2-Year Diet Intervention	No measure of automaticity
13	Ellingson, L. D.; Lansing, J. E.; DeShaw, K. J.; Peyer, K. L.; Bai, Y.; Perez, M.; Phillips, L. A.; Welk, G. J.	2019	Evaluating Motivational Interviewing and Habit Formation to Enhance the Effect of Activity Trackers on Healthy Adults' Activity Levels: Randomized Intervention	No measure of automaticity
14	Afolabi, T. M.; Pogge, E. K.; Early, N. K.; Larson, S.; Stein, J.; Hanson, L.; Storjohann, T.; Raney, E.; Davis, L. E.	2022	Evaluating the impact of integrating SMART goal setting in preceptor development using the Habits of Preceptors Rubric	Not virtuous health-related habit
15	Friedrich, M.; Fugiel, J.; Goluch, Z.; Bruszkowska, M.	2022	Evaluation of changes in eating habits of chronically mentally ill patients residing in 24-hour social welfare home, subject to various forms of health-promoting education	No measure of automaticity
16	Kaushal, Navin; Rhodes, Ryan	2015	Exercise habit formation in new gym members: a longitudinal study	Not experimental design
17	Lally, P.; Wardle, J.; Gardner, B.	2011	Experiences of habit formation: a qualitative study	Not experimental design
18	Lee, Yoongu; Yoon, Yong-Jin	2019	Exploring the Formation of Exercise Habits with the Latent Growth Model	No focus on habit formation
19	Serieux, E.	2018	Exploring the use of text messages for weight loss and healthy habit formation in St Lucia	Conference abstract
20	Fritz, Heather; Hu, Yi-Ling; Tarraf, Wassim; Patel, Pragnesh	2020	Feasibility of a Habit Formation Intervention to Delay Frailty Progression Among Older African Americans: A Pilot Study	No focus on habit formation

21	Hirschey, R.; Rhodes, R. E.; Kimmick, G.; Pan, W.; Lipkus, I.	2016	Feasibility of an intervention focused on increasing enjoyment of physical activity in order to develop physical activity habits	Conference abstract
22	Kleber, C. J.; Putt, M. S.	1990	Formation of flossing habit using a floss-holding device	Article not found
23	N/A	2000	Forming habits of healthy eating	Not a peer-reviewed journal article
24	Mendoza-Vasconez, A. S.; Badii, N.; Becerra, E. S.; Crespo, N.; Hurst, S.; Larsen, B.; Marcus, B. H.; Arredondo, E. M.	2022	Forming Habits, Overcoming Obstacles, and Setting Realistic Goals: A Qualitative Study of Physical Activity Maintenance Among Latinas	No focus on habit formation
25	Huttunen-Lenz, M.; Hansen, S.; Raben, A.; Westerterp-Plantenga, M.; Macdonald, I.; Stratton, G.; et al.	2022	Forming New Health Behavior Habits During Weight Loss Maintenance—The PREVIEW Study	No measure of automaticity
26	Pirolli, P.	2016	From good intentions to healthy habits: Towards integrated computational models of goal striving and habit formation	No focus on habit formation
27	Verplanken, B.; Faes, S.	1999	Good intentions, bad habits, and effects of forming implementation intentions on healthy eating	No measure of automaticity
28	Tappe, K.; Tarves, E.; Oltarzewski, J.; Frum, D.	2013	Habit formation among regular exercisers at fitness centers: An exploratory study	Not experimental design
29	Harris, M. C.; Kessler, L. M.	2019	Habit formation and activity persistence: Evidence from gym equipment	Not experimental design
30	Danner, U. N.; Aarts, H.; De Vries, N. K.	2007	Habit formation and multiple means to goal attainment: Repeated retrieval of target means causes inhibited access to competitors	Not virtuous health-related habit
31	Hand, G.; Cornelius, T.; Gettens, K.; Leahey, T.; Gorin, A.	2022	Habit Formation in a Couples-Based Weight Loss Intervention	Conference abstract
32	Field, C.; Leahey, T.; Denmat, Z.; O'Connor, K.; Gorin, A.	2022	Habit Formation in an Online Intervention: Impact on Weight Loss and the Role of Executive Function	Conference abstract
33	Iwamoto, K.	2013	Habit formation in household consumption: Evidence from Japanese panel data	Not virtuous health-related habit

34	Mayo-Wilson, L. J.; Devoto, B.; Shelton, A.; MacCarthy, S.; Saya, U.; Linnemayr, S.	2020	Habit formation in support of antiretroviral medication adherence in clinic-enrolled hivinfected adults: A mixed qualitative assessment in Kampala, Uganda	No measure of automaticity
35	Peng, W.; Li, L.; Kononova, A.; Cotten, S.; Kamp, K.; Bowen, M.	2021	Habit formation in wearable activity tracker use among older adults: Qualitative Study	Not experimental design
36	Zhang, C.; Adriaanse, M. A.; Potgieter, R.; Tummers, L.; de Wit, J.; Broersen, J.; de Bruin, M.; Aarts, H.	2022	Habit formation of preventive behaviours during the COVID-19 pandemic: a longitudinal study of physical distancing and hand washing	Not experimental design
37	Weyland, S.; Finne, E.; Krell-Roesch, J.; Jekauc, D.	2020	(How) Does Affect Influence the Formation of Habits in Exercise?	Not experimental design
38	Kaushal, Navin; Rhodes, Ryan; Spence, John; Meldrum, John; Rhodes, Ryan E.; Spence, John C.; Meldrum, John T.	2017	Increasing Physical Activity Through Principles of Habit Formation in New Gym Members: a Randomized Controlled Trial	No measure of automaticity
39	Takai, I.	2013	[Influence of comprehensive intervention composed of nutrition and exercise on the development of exercise habits and self-perceived health among community-dwelling elderly individuals]	No measure of automaticity
40	Luque, D.; Molinero, S.; Watson, P.; López, F. J.; Le Pelley, M. E.	2020	Measuring habit formation through goal-directed response switching	Not virtuous health-related habit
41	John, C. E.; McCracken, C. B.; Haber, S. N.	2010	Motivation on the Mediterranean: reward, compulsions and habit formation	Not virtuous health-related habit
42	Birkbeck, J. A.	1981	Obesity socioeconomic variables and eating habits in new zealand	Article not found
43	Karppinen, Pasi; Oinas-Kukkonen, Harri; Alahaivala, Tuomas; Jokelainen, Terhi; Teeriniemi, Anna-Maria; Salonurmi, Tuire; Savolainen, Markku J.	2018	Opportunities and challenges of behavior change support systems for enhancing habit formation: A qualitative study	No focus on habit formation

44	Smith, C. E.; Dauz, E.; Clements, F.; Werkowitch, M.; Whitman, R.; Smith, Carol E.; Dauz, Emily; Clements, Faye; Werkowitch, Marilyn; Whitman, Robert	2009	Patient education combined in a music and habit-forming intervention for adherence to continuous positive airway (CPAP) prescribed for sleep apnea	No measure of automaticity
45	Fontanet, C. P.; Choudhry, N. K.; Wood, W.; Robertson, T.; Haff, N.; Oran, R.; Sears, E. S.; Kim, E.; Hanken, K.; Barlev, R. A.; Lauffenburger, J. C.; Feldman, C	2021	Randomised controlled trial targeting habit formation to improve medication adherence to daily oral medications in patients with gout	Protocol/methods paper
46	Hussam, R.; Rabbani, A.; Reggiani, G.; Rigol, N.	2022	Rational Habit Formation: Experimental Evidence from Handwashing in India	No measure of automaticity
47	Wu, X.; Ma, Y.	2023	The causes of internal habit formation among Chinese urban residents: a multi-layer model perspective	Not virtuous health-related habit
48	Clarke, G.; Kehoe, J.; O'Broin, D.	2017	The effects of gamification on the formation of a habit of studying in tertiary level students	Not a peer-reviewed journal article
49	Feldhacker, Diana R.	2022	The feasibility of an occupation-based habit formation intervention in promoting healthy behaviors among individuals with type 2 diabetes: A mixed methods study	Thesis
50	Putt, M.; Reese, P.; Barankay, I.; Russell, L.; Pagnotti, D.; McGillovary, R.; Finnerty, D.; Chada, S.; Hoffer, K.; Volpp, K.	2018	The habit formation trial to evaluate behavioral economic interventions to reduce the risk of cardiovascular disease: Lessons learned	Conference abstract
51	McCaul, K. D.; Glasgow, R. E.; O'Neill, H. K.	1992	The problem of creating habits: establishing health-protective dental behaviors	No measure of automaticity
52	Benadof, Dafna	2016	Tooth brushing habit formation in children of Mexican immigrant families in Pennsylvania, U.S.: A qualitative study	Thesis
53	Khinda, R.; Parameswaran, S.; Mitra, G.; Lin, X.; Verni, C.; Kishore, R.; Billittier, A.	2017	Use of gamified social media with home telemonitoring for patient self-management in poorly controlled medicaid diabetics: A pilot study of health outcomes, social influences, and habit formation	No measure of automaticity

54	Pagoto, S.; Tulu, B.; Agu, E.; Waring, M. E.; Oleski, J. L.; Jake-Schoffman, D. E.	2018	Using the Habit App for Weight Loss Problem Solving: Development and Feasibility Study	No measure of automaticity

Table S3. Overview of included studies.

Author, year	Study design	Sample description and sample size	Target behaviour	Intervention description	Measure of habit	Results	Determinants of habit formation
Bartle 2019	RT, 2 weeks	n=166 university students and staff<br><br>Female: 71%<br>Male: 29%<br><br>Age: NR	Fruit consumption	Participants were instructed to consume either only apples or any fruit for a two-week period, and given either a generic A4 poster or the opportunity to select a pictorial cue for their poster, primarily fruit-based but including some exceptions. Participants placed their assigned poster on their fridge for the intervention period.	SRHI	Habit strength, mean (SD)<br>Condition 1<br>Baseline: 3.83 (1.56)<br>Post: 4.33 (1.36)<br>Condition 2<br>Baseline: 3.93 (1.21)<br>Post: 4.31 (1.18)<br>Condition 3<br>Baseline: 3.62 (1.31)<br>Post: 4.12 (1.23)<br>Condition 4<br>Baseline: 3.74 (1.41)<br>Post: 4.26 (1.20)	NR
Beeken, 2017	RCT, 24 months	n=537 adults with obesity<br><br>Female: 66%<br>Male: 34%<br><br>Age: median (IQR), 59.4 (48.7–66.8)	Diet and physical activity for weight loss	Ten Top Tips: self-guided program using a leaflet based on habit-formation theory to encourage negative energy balance behaviours. Health professionals, trained to deliver the intervention consistently, provided the leaflet, a logbook for tracking behaviours and weight, and a guidance card during a 30-minute session. Compliance was ensured through quality checks, and supplementary materials were made available for standardization and support. Additionally, patients received various forms of lifestyle advice and community referrals, such as gym prescriptions, dietitian referrals, weight management programs, and support groups like Weight Watchers and Slimming World, tailored to their needs based on different primary care practices (PCPs).	SRHI	Change in automaticity, mean (SD):<br>Intervention: 19.5 (21)<br>Control: 26.9 (22.4)<br><br>Greater automaticity in intervention group compared with control group (adjusted difference = 8.45; 95% CI = 2.59, 14.32).	NR
Cleo 2019	RCT, 12 weeks	n=75 adults<br><br>Female: 72%<br>Male: 28%<br><br>Mean: 50.5 yo	Diet and physical activity	Ten Top Tips: focusing on 7 behaviours for negative energy balance, two for food intake awareness, and one for routine promotion, supported by self-monitoring through a logbook after a 2-hour induction meeting.<br>Do Something Different: daily activities tracked online, tailored to individual habits, supported by weekly calls and monitored via logbooks and online software, a 2-hour induction meeting and ongoing support.	12-item habit rater questionnaire	Habits score, mean change (95% CI) from baseline:<br>TTT: −1.9 (−3.0, −0.7)<br>DSD: 0.4 (−0.7, 1.5)<br>WL: 0.5 (−0.9, 1.8)	NR
Davis 2020	Single group pre-post, 12 weeks	n=100 hospital patients<br><br>Female: 96%<br>Male: 4%<br><br>20–40 yo: 21%<br>41–60 yo: 57%	Physical activity	Technology-based 12-week PA habit development program that included wearable technology, tracked step counts, daily text messaging, and weekly electronic newsletters.	SRHI	Strong habit of desired activity, n (%):<br>Week 1: n=0<br>Week 12: n=20 (61%)<br><br>Mean (SD) not reported.	NR

		60+ yo: 14%<br>NR: 8%					
Diefenbacher 2023	Single group pre-post, 5 weeks	n=58 general public<br><br>Female: 39.7%<br>Male: 58.6%<br>NR: 1.7%<br><br>Mean (SD): 35.3 (11.0) yo	Fruit consumption	Participants selected a specific meal as a cue for a new habit of daily fruit consumption, forming implementation intentions by associating the chosen mealtime with eating one piece of fruit and reinforcing it through image selection, while also maintaining a daily record of context-specific and context-free fruit consumption over 3 weeks.	SRBAI	SRBAI<br>Context-specific habit score, mean (SD)<br>Timepoint 1: 2.77 (1.77)<br>Timepoint 4: 3.27 (1.73), p=0.060<br><br>Context-free habit score, mean (SD):<br>Timepoint 1: 4.00 (1.56)<br>Timepoint 5: 4.16 (1.67), p=1.00	Context
Fournier 2017	RCT, 90 days	n=48 university students<br><br>Female: 42%<br>Male: 58%<br><br>Mean (SD): 21.7 (1.8) yo	Psoas iliac stretch	Participants performed a daily stretching exercise for 90 days, focusing on the recommended psoas iliac stretch to maintain flexibility and prevent low back pain. The timing of the stretch varied based on group allocation, either in the morning upon waking or before sleeping.	SRBAI<br><br><br><br>Time to reach asymptote (automaticity)	Significant total effect of time of day on habit development (effect = -26.7, SE = 10.2, 95% CI: -47.5, -6.0).<br><br>The behaviour became habitual faster in the morning (105.9 ± 46.7 days) vs. evening (154.0 ± 71.1 days) group.	Timing of habit
Fritz 2022	RCT, 12 weeks	n=20 older African-Americans who were classified as prefrail<br><br>Female: 100%<br><br>Mean (SD): 73.5 (10.0) yo	Reduce Frailty Risk Factors through reducing sitting time and increasing physical activity and modifying dietary patterns	Focused on educating participants about prefrailty, frailty protective behaviours, and habit formation, with sessions addressing pain management, sedentary behaviour reduction, increased physical activity, and dietary modification, concluding with a review of progress, maintenance plans, and treatment closure, employing activity analysis to identify low-complexity behaviours integrated into daily routines and creating personalised habit formation plans linked to recurring situations and environmental modifications, with weekly evaluations of participants' progress.	SRBAI	SRBAI, mean (SD)<br>Baseline: 1.86 (0.87)<br>12 weeks: 4.69 (1.24), p value not reported	NR
Judah 2013	Quasi-experimental, 4 weeks	n=50 university students and general public<br><br>Gender: NR<br><br>Before tooth-brushing group, mean (SD): 26.6 (7.06)<br>After tooth-brushing group, mean (SD): 28.1 (9.12)	Flossing	Participants were given a motivational intervention to promote daily flossing, including persuasive information on its benefits, verbal and written instructions on how and when to floss, and the formation of implementation intentions based on individualised bedtime routines, with participants pledging to floss every night before or after brushing, committing the reminder to memory for a 4-week period.	SRBAI	Automaticity score at 4 weeks, mean (SD)<br><br>Before tooth-brushing group: 19.45 (7.64)<br>After tooth-brushing group: 23.80 (8.59), p=0.075	Participant characteristics<br>Timing of habit
Judah 2018	Single group pre-post, 16 weeks	n=118 general public<br><br>Female: 55%<br>Male: 45%	Flossing and vitamin C tablet adherence	Flossing: a 30–40 minute individual session, receiving an information leaflet and oral explanation detailing the positive health and social consequences of flossing, along with instructions on how and when to floss; participants were guided in forming implementation intentions based on their personal routines, which were	SRBAI	Flossing automaticity, mean (SD)<br>T1: 1.54 (1.55)<br>T4: 3.75 (2.07), p value not reported<br><br>Vitamin C automaticity, mean (SD)<br>T1: 2.11 (1.25)	Pleasure<br>Intrinsic motivation (Vitamin C only)<br>Behaviour rewarding

		Mean (SD): 35.7 (11.8) yo		written on the leaflet, read aloud, and accompanied by a nightly pledge to establish commitment and a behavioral contract.<br><br>Vitamin C: The online vitamin C intervention, embedded in the T0 questionnaire, provided information on vitamin C benefits, assessed participants' perceived benefits, instructed routine-specific vitamin C tablet intake times, and included T1 measures such as multiple-choice questions, reinforcing health consequences awareness, and inquiries about timing, coping planning, reviewing goals, and restructuring the environment.		T4: 3.53 (2.31), p value not reported	
Kaushal, 2018	RT, 8 weeks	n=94 adults<br><br>Female: 77%<br>Male: 23%<br><br>Mean (SD): 40.5 (15.3) yo	Exercise	A workshop and received a follow-up phone call midway through the study. The workshop aimed to help participants develop a preparatory exercise habit by using behaviour change techniques like reward, goal setting, planning, and habit formation. Participants learned about the health benefits of physical activity, how to create enjoyable exercise routines, and strategies for establishing consistent exercise habits, such as setting up cues and creating action plans.	SRBAI	Habit preparation, week 8 mean (SD)<br>Intervention: 3.08 (1.01)<br>Control: 2.62 (1.00)<br><br>Habit performance, week 8 mean (SD)<br>Intervention: 3.61 (.91)<br>Control: 2.91 (1.07)	Affective judgments<br>Behavioural regulation<br>Preparatory habit
Keller 2021	RCT, 12 weeks	n=192 university students and general public<br><br>Female: 86.5%<br>Male: 13.5%<br><br>Mean (SD): 24.76 (7.50) yo	Nutrition behaviour	5-minute online intervention was implemented by health psychologists to promote healthy nutrition behaviours. Both groups received general information and created personalised cue-behaviour plans, with one group focusing on daily routine cues and the other on specific time cues, incorporating behaviour change techniques such as action planning, health consequences awareness, habit formation, and weighing pros and cons. Participants selected novel cue-behavior combinations, followed the planned behavior daily for 12 weeks, and were provided with examples to enhance understanding and adherence to the intervention protocol.	SRBAI<br><br><br><br><br><br><br><br><br><br><br>Time to reach asymptote (automaticity)	SRBAI, mean (SD)<br>Routine condition:<br>Baseline: 2.22 (0.91)<br>Month 3: 3.74 (1.34), p value not reported<br><br>Time condition:<br>Baseline: 2.51 (1.16)<br>Month 3: 3.54 (1.23), p value not reported<br><br>Median of 59 days (range 4 to 335) for participants who successfully formed habits to reach peak automaticity.	Participant characteristics
Kilb 2022	Single group pre-post, 8 weeks	n=253 general public<br><br>Female: 87%<br>Male: 13%<br><br>Mean (SD): 37.1 (13.0) yo	Nutrition behaviour	Participants were guided through a multi-step intervention to establish the higher-order habit of filling half of their dinner plate with vegetables, involving information on health consequences, instructions on recommended vegetable consumption, habit formation principles, selection of contextual cues, awareness of rewards, and the creation of implementation intentions and coping plans, with ongoing self-monitoring through picture submissions to a study WhatsApp account, followed by a summary email upon completion.	SRBAI	Next-day multilevel models for the outcome habit strength,<br>Intercept<br>$\beta = -0.01$<br>SE = (95% CI): 0.04 (-0.08, 0.06)<br>p = 0.798<br><br>Mean (SD) not reported.	Participant characteristics<br>Repetition<br>Context

Lally 2008	RCT, 8 months	n=104 overweight and obese adults<br><br>Female: 66%<br>Male: 33%<br><br>Mean (SD)<br>Wait-list: 36.0 (9.8) yo<br>Monthly weighing intervention: 42.1 (9.9) yo<br>Weekly weighing intervention: 43.3 (11.4) yo	Nutrition behaviour and physical activity	'Ten Top Tips' program: 7 behaviours focused on negative energy balance, 2 aimed at increasing awareness of food intake, and 1 promoting routines, with the goal of creating a daily calorie deficit; the program was designed to be accessible and encourage habit development by incorporating easy language, memorable names, and an attractive leaflet. Participants were instructed to plan and incorporate the tips into their daily routines for the first 8 weeks, with a daily monitoring form provided for self-monitoring and planning adjustments if needed.	SHRI<br><br><br><br><br>Exit survey	SRHI automaticity scores increased by an average of 9 points (on a 42-point scale) after 32 weeks.<br>Mean (SD) not reported.<br><br>n=17 participants self-reported how long it had taken to develop habits: Mean=3.0 months (SD=1.8).	NR
Lally 2010	Single group pre-post, 12 weeks	n=96 university students<br><br>Female: 69%<br>Male: 31%<br><br>Mean (range): 27 (21–45)	Eating, drinking, or exercise behaviour	Participants were instructed, at the initial meeting, to select a healthy eating, drinking, or exercise behaviour that they did not already perform, could be triggered by a daily cue, and had a cue occurring once a day, such as 'eating a piece of fruit with lunch,' 'drinking a bottle of water with lunch,' or 'running for 15 minutes before dinner,' and were then asked to strive to carry out this behaviour daily for 84 days, logging their daily performance on the study website and completing the SRHI.	SHRI<br><br><br>Time to reach asymptote (automaticity)	SHRI mean (SD) not reported.<br><br><br>The median time to reach 95% of automaticity was 66 days (range=18 to 254 days)	Repetition<br>Behaviour type
Mergelsberg 2021	RCT, 3 weeks	n=186 university students and general public<br><br>Female: 78%<br>Male: 22%<br><br>Mean (SD): 28.7 (12.0)	Microwave their dishcloth or sponge for 1 minute/day to reduce the risk of foodborne disease	Received information on microwaving sponges to eliminate bacteria and were randomly assigned to conditions involving cueing (cued or not cued) and monitoring (behaviour monitoring, habit monitoring, or irrelevant behaviour monitoring). Over a three-week intervention, participants received cues and monitoring instructions, followed by post-intervention and follow-up measures, concluding with debriefing one week after the final survey.	SHRI	ANOVA main effect of time on habit strength: $F(1.57,222)=341$, $p<.001$, $\eta^2=0.708$.<br><br>Mean (SD) not reported.<br><br>ANOVA time x sample interaction $F(1.56, 211)=5.57$, p.008, , $\eta^2=0.040$.	Participant characteristics
Mullan 2014	RT, 3 weeks	n=45 university students<br><br>Female: 80%<br>Male: 20%<br><br>Mean (SD): 22.9 (7.5) yo	Microwave their dishcloth or sponge for 1 minute/day to reduce the risk of foodborne disease	Participants informed about microwaving dishcloths and received a sponge. Randomly assigned to three conditions, habit formation groups received a poster for their kitchen. All were informed of upcoming emails for a three-week survey. High-frequency reminders were sent every three days for SRHI completion, low-frequency every five days, and the control group received reminders for a breakfast diary every three days.	SRHI	SRHI change:<br>High frequency: 2.02, $p< 0.01$<br>Low frequency: 3.03, $p<0.01$<br>Control: 1.10, $p=0.03$	NR
Orbell 2010	RT, 14 weeks	n=128 university students<br><br>Female: 53%	Dental flossing	Each participant received a numbered packet of dental floss and completed questionnaires at baseline, 2 weeks, and 4 weeks. After 4 weeks, participants returned the dental floss packets to the researcher.	SRHI	Habit strength, mean (SD)<br>Baseline<br>Intervention: 2.05 (1.44)<br>Control: 1.81 (1.61)	Intention and Implementation Intentions

		Male: 47%<br><br>Mean (SD): 21.15 (1.56)				2 weeks<br>Intervention: 3.37 (1.33)<br>Control: 2.02 (1.69)<br>4 weeks<br>Intervention: 3.76 (1.19)<br>Control: 2.50 (1.56)	
Phillips, 2019	RT, 3 weeks	n=82 healthy adults<br><br>Female: 85%<br>Male: 15%<br><br>Age: NR	Nutrition behaviour	3-minute video on MyPlate Guidelines, set a goal to fill half their dinner plate with fruits and vegetables, and created action and coping plans to achieve this. They were instructed to follow this goal for at least five of seven nights per week for three weeks and to send pictures of their dinner plates three nights each week. The intervention included behaviour change techniques such as self-monitoring, others monitoring, information on health consequences, and planning strategies, while the control group also submitted pictures on the same schedule to ensure comparable monitoring.	SRBAI	Habit strength, mean (SD) at 3 weeks<br><br>Intervention: 4.11 (.70)<br>Control: 3.53 (.93)	NR
Van der Weiden 2020	Single group pre-post, 90 days	n=146 general public<br><br>Female: 81%<br>Male: 19%<br><br>Mean (SD): 31.9 (12.7)	Drinking water<br>Eating vegetables<br>Making contact with others<br>Saving money<br>Being patient with others<br>Paying attention to others<br>Recycling	Participants in the study selected a specific behaviour (e.g., eating fruit, being patient, saving money, recycling) to develop into a habit, choosing from pre-set combinations of behaviours and contexts with a focus on personal relevance and daily feasibility. A mobile app was developed for regular assessments of self-control capacity and habit strength, incorporating daily tests and questionnaires with reminders to support habit formation and self-monitoring throughout the study.	SHRI	Habit strength<br>Linear trend: $t=15.30$, $p<0.001$<br>Quadratic trend: $t=-3.39$, $p<0.001$<br>Mean (SD) of habit strength consistency:<br>Eating fruits: 0.79 (0.27)<br>Eating vegetables: 0.76 (0.27)<br>Drinking water: 0.89 (0.18)<br>Exercise: 0.59 (0.29)<br>Physical activity: 0.78 (0.23)<br>Making contact with others: 0.91 (0.15)<br>Being patient with others: 0.90 (0.14)<br>Paying attention to others: 0.95 (0.07)<br>Saving money: 0.61 (0.33)<br>Recycling: 0.90 (0.14)	Context
White, 2017	RCT, 12 weeks	n=103 older adults<br><br>Female: 59%<br>Male: 41%<br><br>Mean (SD): 68.32 (3.78)	Sedentary behaviour reduction	A printed booklet with information on the health impacts of sedentary behaviour and physical activity, including 15 tips for reducing sedentary behaviour and forming exercise habits. Participants used eight tick-sheets to track daily adherence to these tips, which included light physical activities and strategies to decrease sedentary time. The booklet provided specific cues and behaviours for each tip, along with health-related explanations and optional modifications to suit different activity levels.	SHRI	Sedentary behaviour habit, mean (95% CI) at 12 weeks<br>Intervention: 3.95 (3.66, 4.20)<br>Control: 3.98 (3.64, 4.26)<br><br>Physical activity habit, mean (95% CI) at 12 weeks<br>Intervention: 3.66 (3.34, 3.95)<br>Control: 3.48 (3.14, 3.81)	NR

DSD: Do Something Different intervention; NR: not reported; SD: Standard deviation; SRBAI: The Self-Report Behavioural Automaticity Index; SRHI: Self-report habit index; TTT: Ten Top Tips intervention; WL: waitlist.
